# An Atypical Occurrence of Epidermoid Cyst in the Postauricular Parotid Region of a Young Child: A Case Report and Literature Review

**DOI:** 10.7759/cureus.69784

**Published:** 2024-09-20

**Authors:** Saurabh Tiwari, Debapriya Pradhan, Aishwarya Jethi, Ankit Dhimole, Nikita Saini

**Affiliations:** 1 Department of Pedodontics and Preventive Dentistry, Hitkarini Dental College and Hospital, Jabalpur, IND; 2 Department of Oral Medicine and Radiology, Hitkarini Dental College and Hospital, Jabalpur, IND

**Keywords:** asymptomatic, epidermoid cyst, keratinous material, parotid gland, postauricular region

## Abstract

Benign epidermal cysts, also called epidermoid cysts, can sometimes develop in the orofacial region from trapped epidermal components without skin adnexal appendages. Congenital or acquired conditions could be the case. Epidermoid cysts have been found to occur in 6.9-7% of facial regions. Though these cysts normally grow slowly and present with no symptoms, occasionally a subsequent infection makes them problematic. It's more common in the third and fourth decades of life. It is an uncommon illness among children. This report describes a mass that was seen in the right postauricular region of a nine-year-old child. The lesion was surgically removed, and after achieving hemostasis, it was found to be an epidermoid cyst.

## Introduction

Epidermoid cysts are typically benign, dome-shaped tumors found in the skin that are lined with epithelial cells and filled with thick, degraded epithelial material [1]. They usually appear as elastic, firm nodules with a light blue color and a central black punctate opening. When removed, they often release a white, foul-smelling discharge. Redness, swelling, and tenderness may occur if the cyst ruptures or becomes infected [2]. A rare case involved an epidermoid cyst in the postauricular parotid region of a child, which was identified and surgically treated.

## Case presentation

**Figure 1:**
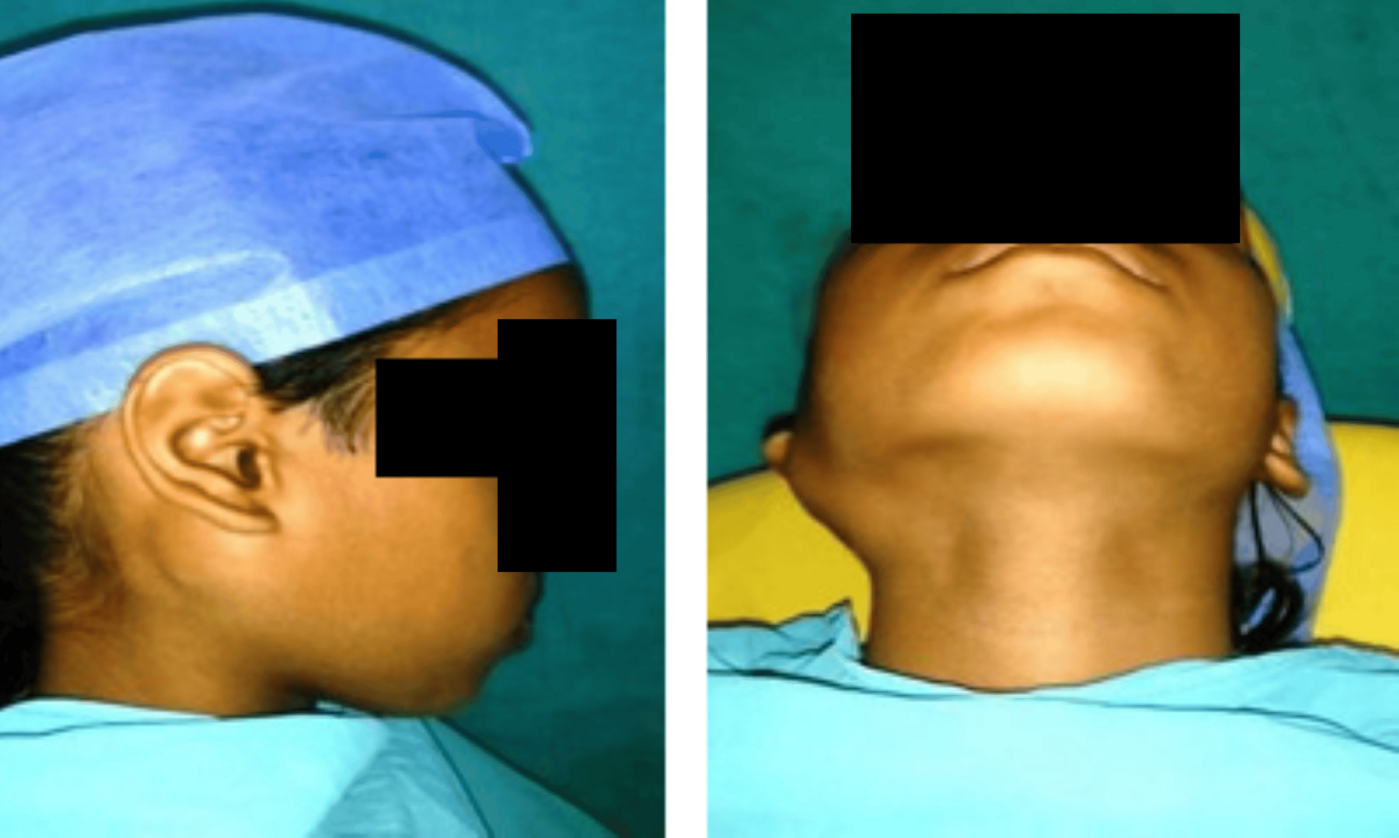
Extraoral view showing the swelling of postauricular parotid region

A nine-year-old girl reported to the Department of Pediatric and Preventive Dentistry with a chief complaint of swelling which was present on the right side of her face. On extraoral examination, a well-demarcated, solitary swelling was found in the postauricular region which was 2.5 × 2.0 cm in dimension (Figure 1).

The overlying mucosa was normal in color and texture without any signs of inflammation. The swelling was firm, non-tender, movable, smooth, and not associated with discharge on palpation. On physical examination, no other swelling was noted elsewhere in the body. There was no associated lymphadenopathy.

The lesion showed free edges with adjacent soft tissue structure. Both temporomandibular joint, left parotid, and bilateral submandibular gland appeared normal. An impression of a well-defined rounded lipomatous lesion at the right parotid region was seen. Ultrasonography report of the right region of the neck showed that the right parotid was normal in size, shape, and echodensity. There was a homogenous area posterior to the parotid and just below the ear lobule which measured 26 × 24 × 36 mm. Routine laboratory investigations were normal which included complete blood count, liver function, and renal function.

**Figure 2:**
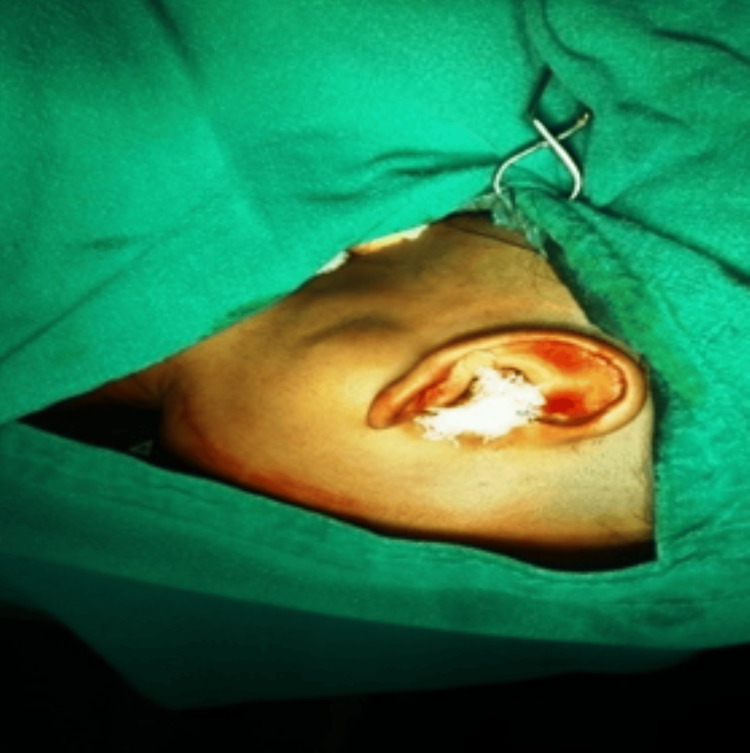
Preparation of surgical site

The cyst was removed by surgical excision under general anesthesia and local anesthesia was given in the postauricular parotid region (Figure 2).

**Figure 3:**
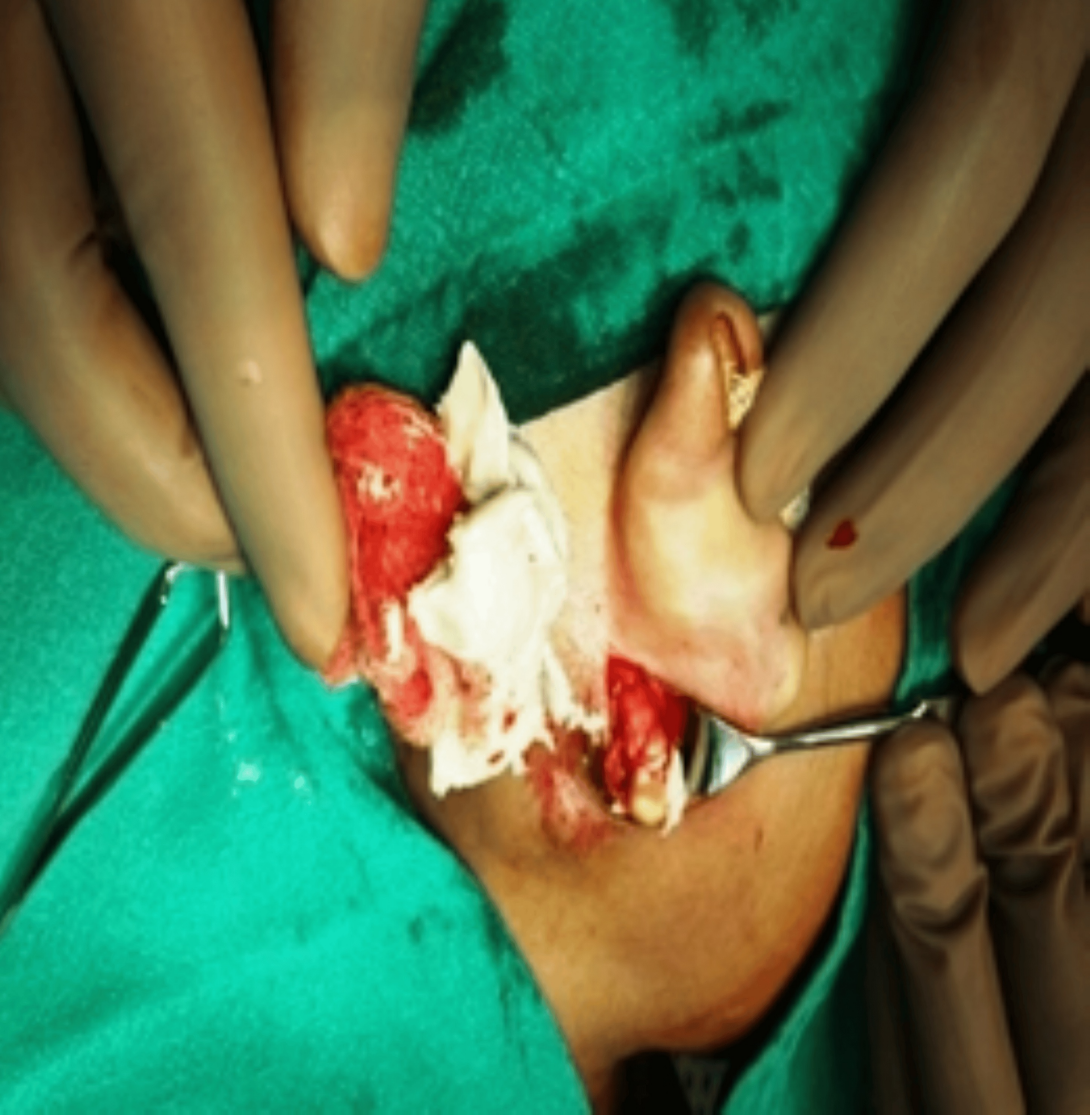
White, gruel-like discharge with capsule

A horizontal incision was placed with BP blade no. 15 above the parotid region, dissected around the capsule of mass. When pressed after excision, the cyst exuded a putrid-smelling, white, gruel-like discharge (Figure 3).

**Figure 4:**
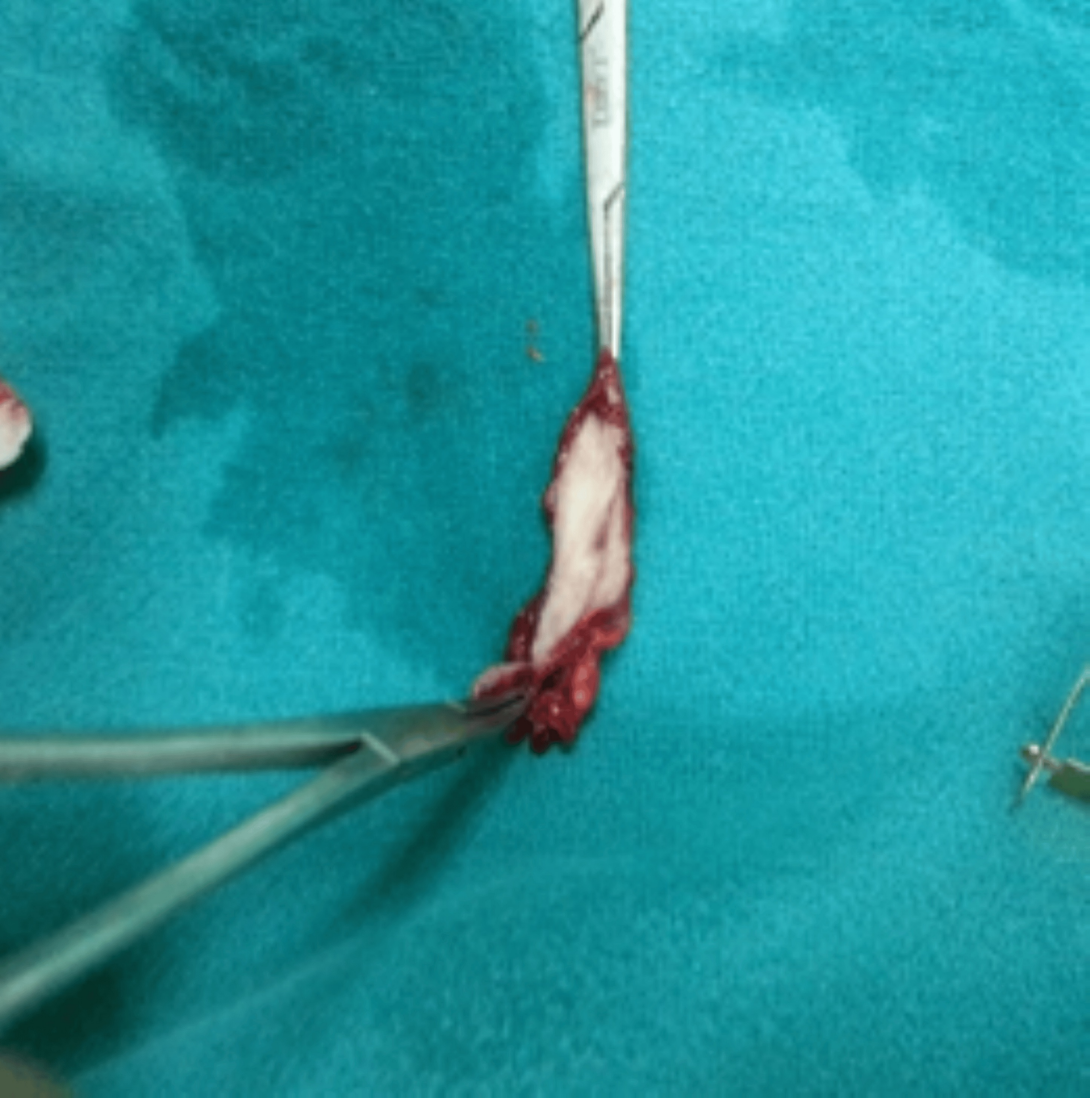
Intact capsule after removal

The mass was resected enblock with an intact capsule which measured about 2.5 × 3.0 cm and weighed 4 g (Figure 4).

**Figure 5:**
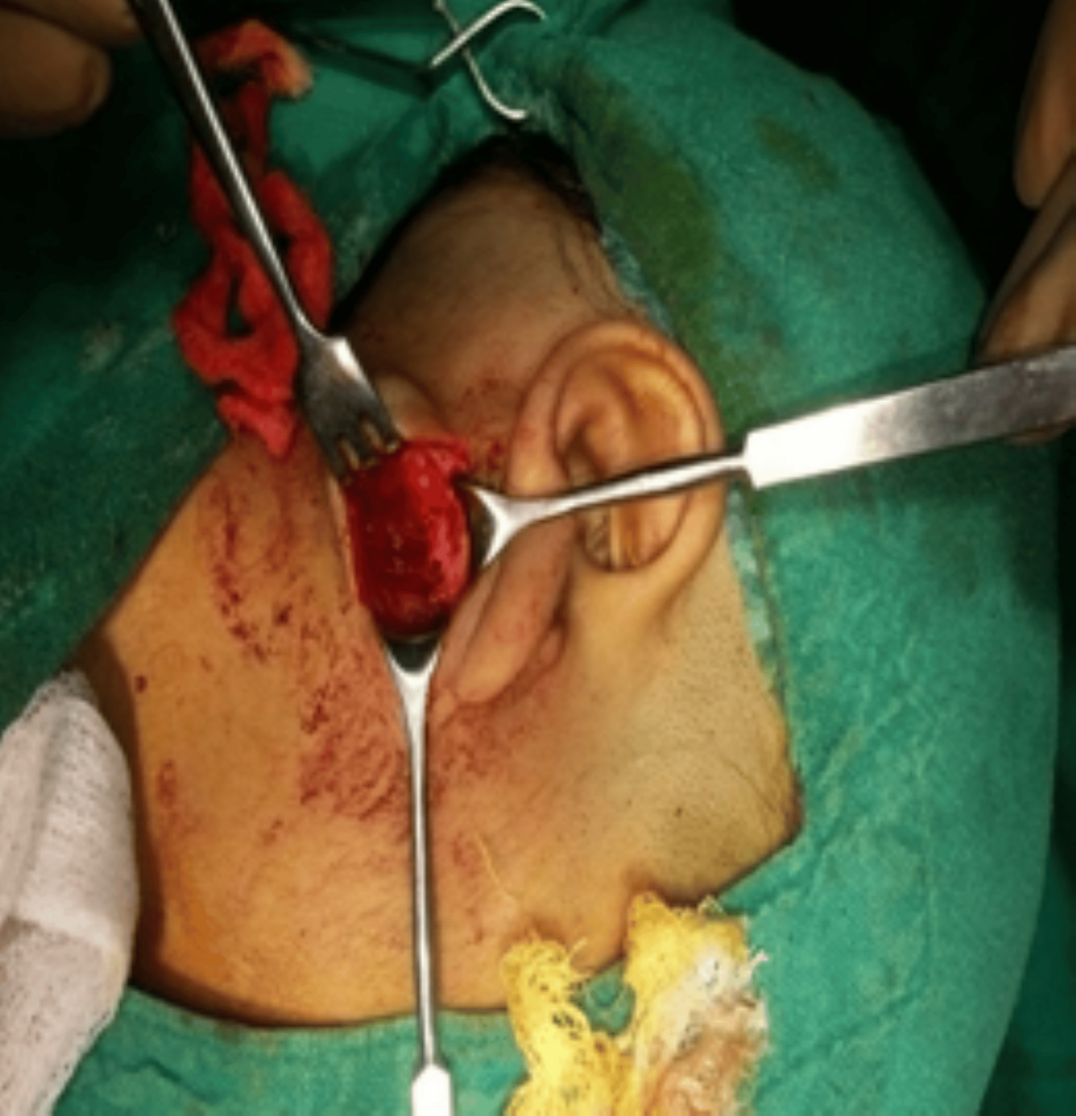
Area after irrigation

**Figure 6:**
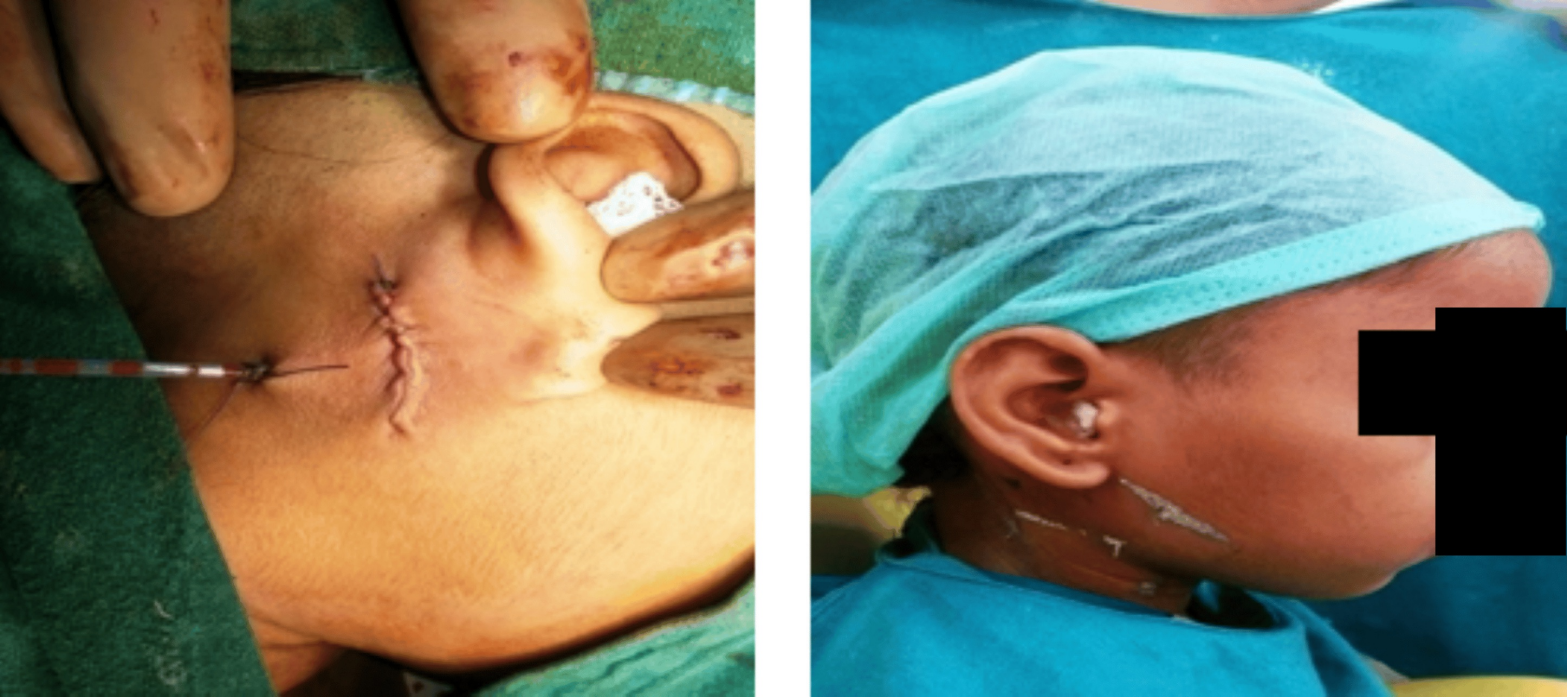
Closure of incision site by suture placement

The area was then irrigated (Figure 5) followed by the closure of the incision site by layer-by-layer vicryl absorbable suture in the inner and subcuticular suture No. 3-0 was placed on the outer surface layer (Figure 6).

On aspiration, cheesy material came out. Fine needle aspiration cytology (FNAC) microscopic investigation of the smear showed good cellularity and showed the presence of numerous anucleate squames and few mature squamous cells against the dirty background. These features were suggestive of a benign cystic lesion-epidermoid cyst.

## Discussion

A cyst is defined as a pathological cavity filled with fluid, which can be solid, semisolid, or gaseous, and may or may not be lined by epithelial cells. An epidermoid cyst specifically has an epidermal lining with a granular layer but lacks glandular cells and is filled with keratinous material [3]. Typically, an epidermoid cyst appears as a slow-growing, asymptomatic, dome-shaped mass that feels fluctuating to firm and is not attached to the underlying structures. These cysts are commonly found in hair-bearing areas and are usually solitary, though multiple cysts can occur. Most epidermoid cysts measure between 1 cm and 5 cm in diameter and are unilocular. While they are usually an isolated occurrence, they can be associated with certain hereditary conditions such as Gorlin syndrome, Gardner syndrome, and Lowe syndrome. If an epidermoid cyst ruptures spontaneously or due to trauma, it can release keratinous material, leading to redness, swelling, pain, and an unpleasant odor. Secondary infection of the cyst can result in abscess formation. In very rare cases, epidermoid cysts may develop into squamous cell carcinoma, basal cell carcinoma, Bowen’s disease, melanoma, or mycosis fungoides [4]. Typically, epidermoid cysts are benign and slow-growing, most often found in younger individuals [5]. Sudden enlargement of the cyst could suggest an infection or malignancy [6]. Squamous cell carcinoma originating from an epidermoid cyst, though rare, has been documented in 10 cases [7,8]. The primary cause of epidermoid cysts is often related to follicular infundibulum, traumatic implantation, or the entrapment of epithelial remnants during embryonic development, while dermoid cysts are caused by the entrapment of epithelium during developmental stages [9]. The distinction between epidermoid and dermoid cysts is based on histopathological findings, particularly the presence or absence of skin appendages in the cyst wall [10]. Few cases of epidermoid cysts in the head and neck region of pediatric patients have been reported in oral and maxillofacial surgery literature (Table 1). A search in PubMed revealed only one case of a postauricular dermoid cyst in a pediatric patient. This report presents a rare case of surgical management of a postauricular epidermoid cyst with involvement of the parotid gland in a child.

**Table 1:** Details of epidermoid cyst occurring on head and neck region in children reported in literature

Author	Year	Location
Jihye, Namki, Seonmi [1]	2020	Inner mucosa of the upper lip
Yavuz [11]	2017	Sublingual
Gary [12]	2015	Postauricular temporal bone
Nasim, Perumal [13]	2014	Floor of the mouth
Kaitlyn [14]	2014	Soft palate
Enver [15]	2003	Pterygopalatine fossa
Midion [16]	1988	Submental region

## Conclusions

This case involved a rare occurrence of an epidermoid cyst located in the postauricular parotid region. The diagnosis was confirmed through a CT scan and histopathological analysis, and the cyst was treated surgically. Although epidermoid cysts are uncommon in young children, they can still appear in this age group. It is important for clinicians to consider this possibility when making a differential diagnosis. Pediatric dentists often identify pathologies both inside and outside the oral cavity, so any extraoral swelling detected during oral examinations should be thoroughly assessed.
